# Risk factors for pelvic organ prolapse in postpartum women: a retrospective cross-sectional study in Southwest China

**DOI:** 10.3389/fmed.2025.1663043

**Published:** 2025-10-07

**Authors:** Dehua Wan, Taizhou Qin, Ling Guo, Xueping Zhang, Huarong Wang, Zhongyan Zheng, Xiaoqin Gan, Tianjiao Liu, Yonghong Lin

**Affiliations:** ^1^Guidance Center of Women's Healthcare Center, Chengdu Women's and Children's Central Hospital, School of Medicine, University of Electronic Science and Technology of China, Chengdu, China; ^2^Department of Gynecology, Chengdu Women's and Children's Central Hospital, School of Medicine, University of Electronic Science and Technology of China, Chengdu, China

**Keywords:** pelvic organ prolapse (POP), risk factor, gestational weight gain, postpartum, pelvic floor dysfunction, forceps delivery, advanced maternal age

## Abstract

**Background:**

Pelvic organ prolapse (POP) is prevalent among postpartum women and can have detrimental effects on their urinary, sexual, and mental well-being. With recent shifts in birth policy and increasing parity in China, the risk of POP among postpartum women is rising. However, large-scale studies focusing on perinatal predictors of POP in Chinese population remain limited.

**Methods:**

We conducted a retrospective study of 8,565 postpartum women who delivered at Chengdu Women’s and Children’s Central Hospital between January 2019 and April 2025. Demographic and perinatal characteristics were collected and pelvic floor function was assessed at sixth week postpartum. POP was diagnosed based on result of the POP-Q system, physical and ultrasound examination, and clinical assessment. Multinomial logistic regression analyses were performed to identify risk factors associated with POP.

**Results:**

The overall prevalence of POP was 72.83%, with most cases classified as Stage I. Occupational type, vaginal delivery, higher parity, and advancing maternal age were significantly associated with the occurrence of POP. Compared with white-collar workers, housewives had a reduced risk of POP (OR = 0.89, 95% CI: 0.81–0.98), whereas blue-collar workers showed no significant difference. Cesarean section was protective (OR = 0.14, 95% CI: 0.12–0.16, *p* < 0.001). High pregestational BMI was associated with a higher risk of POP, while gestational weight gain showed no significant association. Neonatal birth weight did not correlate with POP.

**Conclusion:**

Vaginal delivery, multiparity, advanced maternal age, and high pregestational BMI are risk factors for POP. Perinatal risk assessment and the potential value of preventive strategies, including weight management and individualized delivery planning are essential for mitigating the risks of postpartum POP.

## Background

Pelvic organ prolapse (POP) refers to the descent or displacement of pelvic organs, such as the bladder, uterus, rectum, or small intestine, due to weakening or failure of the fibromuscular support structures, resulting in abnormal positioning within or outside the vaginal canal (1, 2). POP can negatively impact multiple aspects of women’s lives, including urinary function, sexual health, and mental well-being, with psychosocial impacts often manifesting as feelings of loneliness, social isolation, inadequacy, imperfection, and shame (3–5). A study conducted in the United States in 2000 reported that approximately 50% of women undergoing routine gynecological examinations were affected by POP (6). A population-based epidemiological study conducted in China during the 2010s reported a 9.6% nationwide prevalence of symptomatic POP among women aged over 20 years old (7).

In recent years, although China has experienced a decline in overall fertility rates, changes in national birth policies have led to an increasing proportion of second and third births. In 2022, among the 9.56 million live births recorded in China, approximately 40% were second births, while around 15% were third births or higher-order births (8). As parity is the independent risk factor of POP, the incidence of POP among Chinese parous women appears to be on the rise in recent decades (9). Previous studies also have identified other key risk factors for POP, including mode of delivery, advancing age, and obesity (10). These findings suggest that perinatal factors are closely associated with the development of POP. However, few large-scale studies have specifically focused on postpartum women and perinatal characteristics to determine which factors predicts the occurrence of POP.

This study represents the one of the largest large-scale investigations to date assessing POP at 6th postpartum week in China (11, 12). We analyzed the demographic and perinatal characteristics of over 8,500 postpartum Chinese women in a five-year study period, in order to identify perinatal risk factors for POP and to inform clinical management during pregnancy to reduce the risk or improve the prognosis of POP in postpartum women.

## Method

### Study design

**Figure 1:**
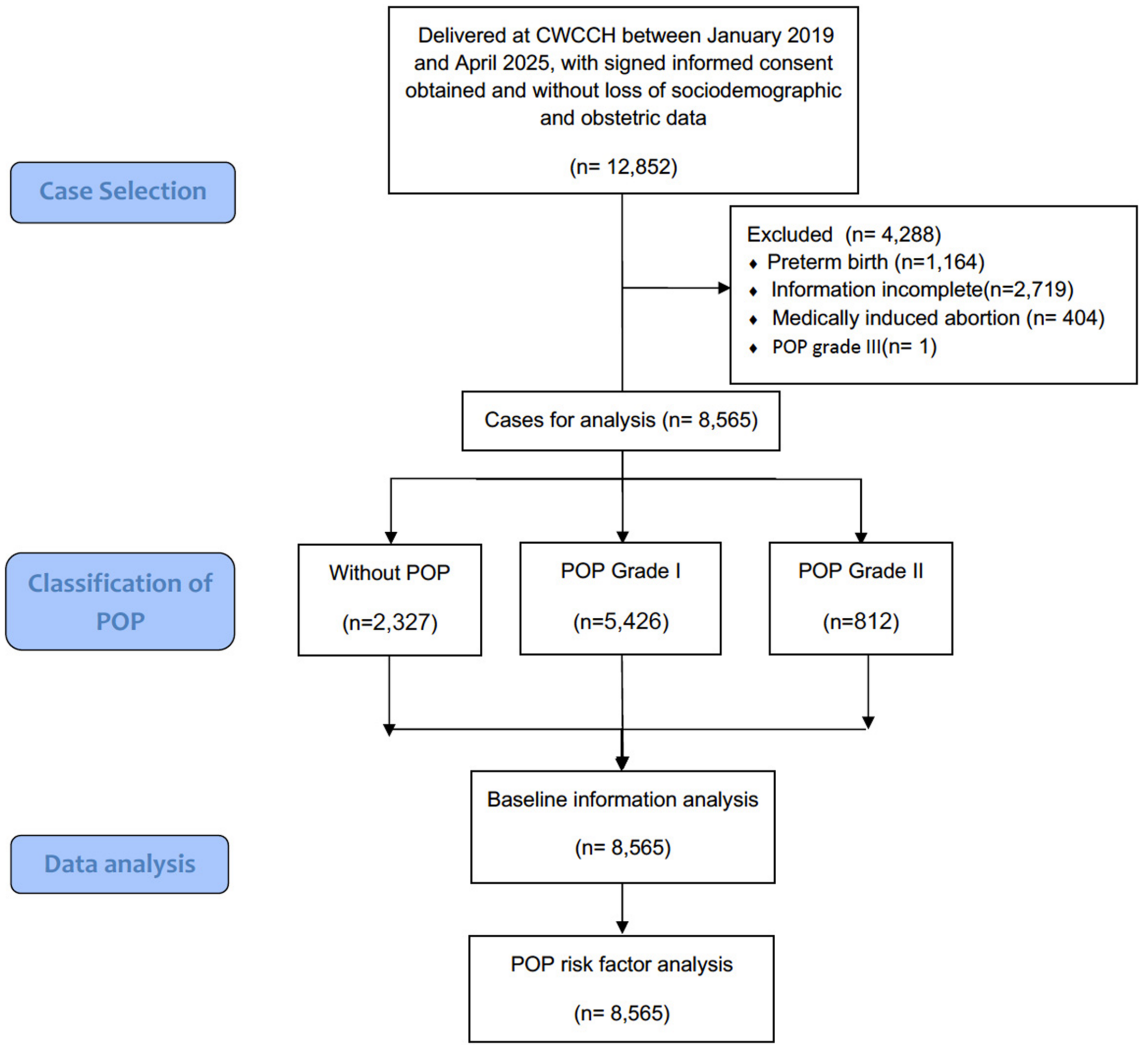
Diagram flowchart of case selection and study analysis.

This retrospective cross-sectional study included 8,565 postpartum women with complete sociodemographic and obstetric data who delivered at Chengdu Women’s and Children’s Central Hospital, a tertiary university-affiliated maternal and child health hospital with an annual delivery volume of 15,000 to 20,000 that serves as a referral center for Chengdu and its surrounding areas, covering an estimated population of approximately 20 million, between January 2019 and April 2025. The diagram flowchart of case selection and study analysis was presented in Figure 1. Cases involving preterm birth (before 37 weeks of gestation) and medically induced abortion were excluded from the analysis. Additionally, since there was only one case of stage III POP in the entire cohort, it was also excluded to facilitate subsequent statistical analyses and data presentation. The demographic and perinatal characteristics of the study cohort were systematically analyzed. To investigate the association between these variables and the development of POP, multinomial ordered logistic regression model was established to identify specific obstetric and demographic characteristics significantly correlated to the occurrence of POP.

### Diagnosis and classification of POP

At our institute, postpartum pelvic floor pressure assessment is routinely conducted at approximately sixth week postpartum, coinciding with the standard postnatal check-up. The diagnostic and staging criteria for pelvic organ prolapse (POP) in this study are based on the guidelines outlined in *Pelvic Organ Prolapse: ACOG Practice Bulletin Summary, Number 214* the *Chinese Guidelines for the Diagnosis and Treatment of Pelvic Organ Prolapse* (2020 Edition) and *the International Continence Society/International Urogynecology Association Pelvic Organ Prolapse Quantification (POP-Q)* system (13–18). The classification is determined by the furthest extent of prolapse in relation to the hymen, across one or more compartments (19, 20).

Stage 0: No evidence of prolapse; the anterior and posterior landmarks are all situated at −3 cm, and point C or D lies between–TVL and –(TVL–2) cm.
Stage I: Criteria for stage 0 are not satisfied, and the leading edge of prolapse remains more than 1 cm above the hymenal plane (i.e., less than −1 cm).
Stage II: The lowest point of prolapse is located within 1 cm above to 1 cm below the hymen (i.e., any point between–1 cm, 0, or +1 cm).
Stage III: The prolapsed tissue extends more than 1 cm beyond the hymen but does not exceed TVL minus 2 cm in descent.
Stage IV: Indicates full vaginal eversion or total prolapse (procidentia), with the furthest point extending to at least (TVL–2) cm.

### Statistical analysis

This study collected a range of perinatal and demographic variables, including maternal age, occupational type (blue-collar work, white collar work, or housewives), body mass index (BMI), fetal birth weight, gestational weight gain (kg), gravidity, parity, mode of delivery, and forceps delivery. Data processing and statistical analyses were performed using SPSS version 27.0 (IBM Corp., Armonk, NY, United States) and Microsoft Excel (Microsoft Corp., Redmond, WA, United States). Categorical variables are summarized as frequencies and percentages and compared using the Mann–Whitney U test or Spearman rank correlation test. Variables that demonstrated statistically significant difference in the univariate analysis and factors potentially relevant to the occurrence of POP were subsequently analyzed in a multinomial ordered logistic regression model to identify independent perinatal and demographic predictors associated with the occurrence of POP. A two-tailed *p*-value of <0.05 was considered statistically significant.

## Results

### Participant demographics and overall prevalence of pelvic organ prolapse

Table 1 presents the demographic characteristics of the study population along with the overall prevalence of POP. Among the 8,565 postpartum women included in the analysis, 2,327 (27.17%) had no POP, 5,426 (63.35%) were classified as stage I, and 812 (9.48%) as stage II, resulting in an overall POP prevalence of 72.83% (95% Confidence interval (CI), 72.26–73.39%). The majority were aged 30–39 years (*n* = 4,617, 53.91%), followed by those under 30 years (*n* = 3,815, 44.55%), while only a small proportion were aged 40 or older (*n* = 132, 1.54%). Regarding occupation, more than half of the participants were white-collar workers (*n* = 4,736, 55.3%), 849 (9.91%) were blue-collar workers, and the remainder were housewives. In terms of BMI, the majority of women (*n* = 5,665, 66.14%) had a BMI between 18.5 and 23.9 kg/m^2^, 2,208 (25.79%) were overweight (BMI 24.0–27.9 kg/m^2^), and 352 (4.11%) were classified as obese (BMI ≥ 28.0 kg/m^2^). Underweight women (BMI < 18.5 kg/m^2^) accounted for 340 cases (3.97%).

**Table 1:** Overall demographic characteristics and prevalence of POP in the study cohort.

Characteristic	Subgroup	*N*	Proportion (%)
Age group (years)	20–29	3,816	44.55
	30–39	4,617	53.91
	≥40	132	1.54
Occupation	While collar work	4,737	55.31
	Blue collar work	849	9.91
	Housewife	2,979	34.78
BMI (kg/m^2^)	<18.5	339	3.96
	18.5 ≤ BMI<24.0	5,665	66.14
	24.0 ≤ BMI<28.0	2,209	25.79
	BMI ≥ 28.0	352	4.11
POP prevalence	None	2,327	27.17
	Stage I	5,426	63.35
	Stage II	812	9.48
	Overall	6,238	72.83
Total		8,565	100

### Anatomical distribution of prolapse across vaginal compartments

Table 2 shows the distribution of prolapsed organ sites among postpartum women by POP-Q stage. Overall, 2,327 women (27.17%) had no POP. Among those affected, the anterior vaginal wall was the most common site, involved in 5,373 women (62.73%) with stage I and 799 (9.33%) with stage II. Posterior vaginal wall prolapse occurred in 1,534 women (17.91%) with stage I and 45 (0.53%) with stage II, while uterine prolapse was less frequent, observed in 433 (5.06%) with stage I and only 2 (0.02%) with stage II. In total, 5,426 women (63.35%) had stage I and 812 (9.48%) had stage II POP.

**Table 2:** Distribution of prolapsed organ sites among postpartum women by POP-Q stage.

POP site	Without POP (*N*, %)	Stage I (*N*, %)	Stage II (*N*, %)
Anterior vaginal wall	–	5,373 (62.73%)	799 (9.33%)
Posterior vaginal wall	–	1,534 (17.91%)	45 (0.53%)
Uterus	–	433 (5.06%)	2 (0.02%)
Overall	2,327 (27.17%)^a^	5,426 (63.35%)^b^	812 (9.48%)^c^

a Represents women without POP.

b Represents women with at least one site of POP, with the maximum POP-Q stage being stage I.

c Represents women with at least one site of POP stage II.

### Univariate analysis on sociodemographic and perinatal characteristics in postpartum women with different classifications of POP

Table 3 presents the results of a comparative analysis of sociodemographic and perinatal characteristics among postpartum women with different severity of POP. Among women younger than 30 years, 72.88% had POP (63.99% stage I and 8.88% stage II). In the 30–39 years group, the prevalence was 72.69% (62.81% stage I and 9.88% stage II). In women aged ≥40 years, the prevalence reached 76.52%, with 63.64% stage I and 12.88% stage II. However, the overall differences among age groups were not statistically significant (*p* = 0.405). The prevalence of POP was comparable across occupational groups (*p* = 0.188). Among white-collar workers (*n* = 4,737), 73.19% had POP, including 63.69% with stage I and 9.50% with stage II. Blue-collar workers (*n* = 849) showed a prevalence of 74.8%, with 63.02% stage I and 11.8% stage II. Housewives (*n* = 2,979) had a prevalence of 71.70%, with 62.91% stage I and 8.79% stage II. The distribution of POP across BMI categories showed no statistically significant differences (*p* = 0.129). For women with BMI < 18.5 kg/m^2^ (*n* = 339), the prevalence of POP was 69.91% (63.13% stage I and 6.78% stage II). In the normal weight group (18.5 ≤ BMI < 24.0, *n* = 5,665), prevalence was 72.66% (63.48% stage I and 9.18% stage II). Among overweight women (24.0 ≤ BMI < 28.0, *n* = 2,209), prevalence was 73.52% (63.11% stage I and 10.41% stage II). In the obese group (BMI ≥ 28.0, *n* = 352), prevalence reached 74.15% (63.07% stage I and 11.08% stage II). Parity’s distribution was also significantly different across different POP stages (*p* < 0.001); among women with one birth, 475 out of 6,084 (7.81%) were in Stage II, increasing to 323 out of 2,414 (13.38%) for those with two births and 14 out of 67 (20.90%) among those with three or more births. A similar trend was observed for gravidity (*p* < 0.001), with Stage II POP seen in 303 out of 3,992 (7.59%) among women with one pregnancy, 250 out of 2,390 (10.46%) among those with two, and 259 out of 2,183 (11.86%) among those with three or more. The distribution of neonatal birth weight significantly differed across the POP stages (*p* = 0.003). In the lowest birth weight group (2.5–3.0 kg), the majority of women were classified as POP Stage I (63.94%), with 29.40% in Stage 0 and 6.66% in Stage II. In contrast, the proportion of Stage II increased notably among women whose neonates weighed 3.0–3.5 kg and 3.5–4.0 kg, reaching 10.24 and 10.99%, respectively. Interestingly, in the >4.0 kg group, although Stage II accounted for 8.35%, a higher proportion of women remained in Stage 0 (30.96%) compared to the mid-weight groups. The distribution of POP differed significantly by mode of delivery (*p* < 0.001). Among women who delivered by cesarean section (*n* = 4,732), 1,854 (41.43%) had no POP, 2,515 (56.20%) were classified as stage I, and only 106 (2.37%) as stage II. In contrast, among those who delivered vaginally (*n* = 4,090), only 473 (11.56%) had no POP, while 2,911 (71.16%) were classified as stage I and 706 (17.26%) as stage II. It is noteworthy that women without forceps delivery (*n* = 6,194) had a POP prevalence of 74.20%, with 1,598 (25.80%) having no POP, 3,986 (64.35%) stage I, and 610 (9.85%) stage II. In contrast, those with forceps delivery (*n* = 335) showed a higher overall POP prevalence of 80.90%, with 64 (19.10%) having no POP, 236 (70.45%) stage I, and 35 (10.45%) stage II. This difference was statistically significant (*p* = 0.017).

**Table 3:** Comparison of the sociodemographic and perinatal characteristics of postpartum women with different POP stages.

Variable	Subgroup	Without POP	Stage I (%)	Stage II (%)	Overall POP (%)	*P*-value
Age (years)	<30	1,035 (27.13)	2,442 (63.99)	339 (8.88)	2,781 (72.88)	0.405^a^
	30–39	1,261 (27.31)	2,900 (62.81)	456 (9.88)	3,356 (72.69)	
	≥40	31 (23.48)	84 (63.64)	17 (12.88)	101 (76.52)	
Occupation	While collar work	1,270 (26.81)	3,017 (63.69)	450 (9.50)	3,467 (73.19)	0.188^b^
	Blue collar work	214 (25.21)	535 (63.02)	100 (11.77)	635 (74.79)	
	Housewife	843 (28.30)	1874 (62.91)	262 (8.79)	2,136 (71.70)	
BMI (kg/m^2^)	<18.5	102 (30.09)	214 (63.13)	23 (6.78)	237 (69.91)	0.129^a^
	18.5 ≤ BMI<24.0	1,549 (27.34)	3,596 (63.48)	520 (9.18)	4,116 (72.66)	
	24.0 ≤ BMI<28.0	585 (26.48)	1,394 (63.11)	230 (10.41)	1,624 (73.52)	
	BMI ≥ 28.0	91 (25.85)	222 (63.07)	39 (11.08)	261 (74.15)	
Neonatal BW (kg)	2.5 ≤ BW<3.0	596 (29.40)	1,296 (63.94)	135 (6.66)	1,431 (70.60)	0.003^a^
	3.0 ≤ BW<3.5	1,068 (26.27)	2,581 (63.49)	416 (10.24)	2,997 (73.73)	
	3.5 ≤ BW<3.9	537 (25.99)	1,302 (63.02)	227 (10.99)	1,529 (74.01)	
	>4.0	126 (30.96)	247 (60.69)	34 (8.35)	281 (69.04)	
Gestational weight gain (kg)	<15	1777 (27.57)	4,050 (62.83)	619 (9.60)	4,669 (72.43)	0.372^b^
	≥15	550 (25.96)	1,376 (64.94)	193 (9.10)	1,569 (74.04)	
Gravidity	1	1,132 (28.36)	2,557 (64.05)	303 (7.59)	2,860 (71.64)	<0.001^a^
	2	615 (25.73)	1,525 (63.81)	250 (10.46)	1775 (74.27)	
	≥3	580 (26.57)	1,344 (61.57)	259 (11.86)	1,603 (73.43)	
Parity	1	1731 (28.45)	3,878 (63.74)	475 (7.81)	4,353 (71.55)	<0.001^a^
	2	584 (24.19)	1,507 (62.43)	323 (13.38)	1830 (75.81)	
	≥3	12 (17.91)	41 (61.19)	14 (20.90)	55 (82.09)	
Delivery mode	Cesarean section	1854 (41.43)	2,515 (56.20)	106 (2.37)	2,621 (58.57)	<0.001^b^
	Vaginal delivery	473 (11.56)	2,911 (71.16)	706 (17.26)	3,617 (88.44)	
Forceps delivery	No	1,598 (25.80)	3,986 (64.35)	610 (9.85)	4,596 (74.20)	0.017^b^
	Yes	64 (19.10)	236 (70.45)	35 (10.45)	271 (80.90)	

a Mann–Whitney U test,

b Spearman’s rank correlation analysis.

BW, birth weight.

### Correlation analysis between the sociodemographic and perinatal characteristics and the occurrence of POP

The multinomial ordered logistic regression analysis identified several independent factors significantly associated with the occurrence of POP (Table 4). Women aged 20–29 years had a significantly lower risk of POP compared to those aged ≥40 years (OR = 0.56, 95% CI: 0.34–0.93, *p* = 0.025). Relative to white-collar workers, housewives demonstrated a significantly lower risk of POP (OR = 0.89, 95% CI: 0.81–0.98, *p* = 0.018), while no significant association was observed for blue-collar workers. When compared with the obese group (BMI ≥ 28.0 kg/m^2^), women with BMI < 18.5 kg/m^2^ were significantly less likely to experience POP (OR = 0.59, 95% CI: 0.38–0.92, *p* = 0.019). Women with normal weight (18.5 ≤ BMI < 24.0 kg/m^2^) also showed a trend toward reduced risk (OR = 0.73, 95% CI: 0.53–1.01, *p* = 0.058), although this did not reach statistical significance. In contrast, overweight women (24.0 ≤ BMI < 28.0 kg/m^2^) demonstrated no significant association with POP risk (OR = 0.86, 95% CI: 0.62–1.21, *p* = 0.393). Regarding obstetric history, primigravid women showed a reduced risk of POP compared to those with three or more pregnancies (OR = 0.75, 95% CI: 0.61–0.92, *p* = 0.005). Likewise, women with only one childbirth had a significantly lower risk of POP than those with three or more deliveries (OR = 0.49, 95% CI: 0.24–0.99, *p* = 0.048). The mode of delivery was strongly associated with POP risk. Women who underwent cesarean section had a markedly lower risk compared to those who delivered vaginally (OR = 0.14, 95% CI: 0.12–0.16, *p* < 0.001). No statistically significant associations were observed for occupational type, fetal weight, gestational weight gain, forceps delivery.

**Table 4:** Correlation analysis between the sociodemographic and perinatal characteristics and the occurrence of POP.

Variable	Category	Coefficient (B)	95% CI	*P*-value
Age group (years)	20–29	−0.572	0.56 (0.34, 0.93)	0.025
	30–39	−0.444	0.64 (0.39, 1.05)	0.076
	≥40	0^a^		
Occupation	Blue collar work	0.094	1.1 (0.94, 1.28)	0.238
	Housewife	−0.118	0.89 (0.81, 0.98)	0.018
	While collar work	0^a^		
BMI (kg/m^2^)	<18.5	−0.529	0.59 (0.38, 0.92)	0.019
	18.5 ≤ BMI<24.0	−0.312	0.73 (0.53, 1.01)	0.058
	24.0 ≤ BMI<28.0	−0.146	0.86 (0.62, 1.21)	0.393
	BMI ≥ 28.0	0^a^		
Neonatal BW weight (kg)	2.5 ≤ BW<3.0	−0.162	0.85 (0.62, 1.17)	0.316
	3.0 ≤ BW<3.5	−0.065	0.94 (0.69, 1.27)	0.673
	3.5 ≤ BW<3.9	0.063	1.07 (0.78, 1.46)	0.695
	>4.0	0^a^		
Gestational weight gain (kg)	<15	−0.103	0.9 (0.78, 1.04)	0.158
	≥15	0^a^		
Gravidity	1	−0.292	0.75 (0.61, 0.92)	0.005
	2	−0.066	0.94 (0.78, 1.13)	0.482
	≥3	0^a^		
Parity	1	−0.708	0.49 (0.24, 0.99)	0.048
	2	−0.433	0.65 (0.33, 1.29)	0.219
	≥3	0^a^		
Mode of delivery	Cesarean section	−1.968	0.14 (0.12, 0.16)	<0.001
	Vaginal delivery	0^a^		
Forceps delivery	Yes	0.28	1.32 (0.99, 1.77)	0.059
	No	0^a^		

a As reference group.

BMI, body mass index; BW, birth weight; CI, confidence interval.

## Discussion

The reported incidence of POP often varies depending on the study population and the diagnostic criteria applied (7). In our large-scale retrospective cross-sectional study involving 8,565 postpartum women managed in our institute during a half decade period, we observed a notably high prevalence of POP, with 72.83% of participants exhibiting some degree of prolapse, primarily at Stage I (63.35%), slightly lower than the POP prevalence reported by the previous study on the similar population (12). Our findings not only confirmed the substantial burden of pelvic floor dysfunction among postpartum women in our region but also provide some valuable insights into the demographic and obstetric factors associated with POP occurrence and severity.

Parity and vaginal delivery have long been recognized as significant risk factors for the development of POP by some previous studies on foreign population (21). Evidence from previous studies indicates that women who undergo vaginal delivery have a twofold increased risk of developing long-term stress urinary incontinence compared to those who deliver via cesarean section (22). Similar impact of vaginal delivery and parity on the occurrence of POP were also observed in our study. A previous investigation examining the influence of obstetric characteristics on POP in Chinese women during the early postpartum period similarly identified maternal age, delivery mode, perineal trauma, a prolonged or delayed second stage of labor, and fetal macrosomia as contributing factors that may predispose women to POP shortly after childbirth. However, the conclusions of that study are somewhat limited by its modest sample size of only 300 participants (23). Furthermore, a large-scale, nationwide survey of Chinese women aged 20 years and older also found that a history of multiple vaginal deliveries were both associated with significantly increased odds of developing various forms of symptomatic POP (7). Previous studies have demonstrated that women who deliver vaginally exhibit a higher incidence of overall pelvic floor muscle dysfunction, as well as greater impairment of both Type I and Type II muscle fibers, compared to those who undergo cesarean section. Zhao et al. identified vaginal delivery as a significant risk factor for postpartum pelvic floor muscle dysfunction (24). Furthermore, a cross-sectional study evaluating primiparous women within 12 to 24 months postpartum found a clear association between vaginal delivery and levator ani avulsion, whereas no levator ani defects were observed among women who delivered via cesarean section (25).

Previous studies suggested that women who delivered infants with high birth weights had a higher prevalence of pelvic floor muscle weakness compared to those who delivered neonates with either low or normal birth weights (21, 26). This may be attributed to the greater mechanical strain and perineal stretching associated with the passage of larger infants through the birth canal, which can lead to direct trauma or denervation of the pelvic floor muscles, especially during the second stage of vaginal delivery. While a large neonatal head circumference has been recognized as a risk factor for POP due to pelvic muscle injury, it is also strongly correlated with higher birth weight (27, 28). However, in our study, there was no significant correlation between birth weight and the occurrence of POP. This discrepancy between our findings and previous studies may be attributed to the fact that approximately half of the women in our cohort underwent cesarean delivery, which could have influenced the analysis on the association between neonatal birth weight and POP.

The association between instrumental vaginal delivery, including the use of forceps or vacuum extraction and increased risk of pelvic floor trauma remains controversial (21, 29–31). In our study, the incidence of POP was significantly higher among women who underwent forceps-assisted delivery compared to those who delivered spontaneously. Although the *p*-value slightly exceeded the conventional threshold, the trend suggests that forceps delivery may be potentially associated with an elevated risk of POP, as indicated by previous studies (31). The use of forceps or vacuum extraction can lead to excessive stretching or tearing of the levator ani muscle and associated connective tissues, as well as direct nerve injury. Although instrumental delivery may be clinically necessary in certain obstetric scenarios, it remains one of the contributors to postpartum pelvic floor impairment. Preventive strategies, such as optimizing the second stage of labor, careful selection of vaginal and forceps delivery indications are essential to minimize the risk of postpartum POP.

Whether gestational weight gain or pregestational BMI has a negative impact on pelvic floor muscle strength remains debatable. Previous studies have reported conflicting findings about whether higher pregestational BMI or gestational weight gain predispose POP (32–35). In our cohort, though weight gain during pregnancy did not exhibit significant association with the occurrence of POP, pregestational BMI was found a risk factor of POP. Excessive gestational weight gain or higher BMI was reportedly identified as risk factors for decreased type I and type II pelvic floor muscle strength, as well as reduced vaginal dynamic pressure (32, 33, 36–38). The intra-abdominal pressure resulting from increased maternal body mass, or from an already high pregestational BMI, may impose chronic mechanical loading on the pelvic floor, potentially leading to muscle fatigue, tissue laxity, and impaired neuromuscular coordination. Moreover, excessive weight gain is often associated with other risk factors such as fetal macrosomia and prolonged labor, further exacerbating pelvic floor strain (36–38). However, compared to factors such as vaginal delivery, parity, and neonatal head circumference, gestational weight gain and pregestational BMI may have more indirect, chronic, and modest impacts on the development of POP. From this perspective, appropriate weight management during pregnancy seems to be still necessary as part of a comprehensive strategy to prevent postpartum POP.

The relationship between postpartum breastfeeding and the occurrence of POP as well as pelvic floor muscle recovery has been a matter of debate, although it was not included as a follow-up indicator in our study. There is indeed a hypothesis suggesting that postpartum recovery from pelvic floor trauma associated with vaginal delivery may be compromised by the transient hypoestrogenic state during breastfeeding (39). However, it was indicated that breastfeeding after vaginal childbirth is not associated with the development of stress urinary incontinence, POP, or anal incontinence two decades after the first vaginal delivery (39). Moreover, an Israeli study also reported that women with pelvic floor dysfunction (PFD) symptoms prior to or during pregnancy can be reassured that breastfeeding is unlikely to delay pelvic floor recovery (40). We speculate that for POP identified at 42 days postpartum, the short-term hormonal effects may be limited. Moreover, evidence from large randomized controlled trials remains inconclusive regarding the potential benefits of estrogen therapy for postpartum pelvic floor muscle recovery (41–43).

Consistent with many previous studies, we identified age as a significant risk factor for POP (21, 32, 38, 44). Physiologically, advancing age is associated with a decline in overall physical fitness, decreased female sex hormone levels, muscle laxity, and diminished neuromuscular function, all of which can compromise the supportive capacity of the pelvic floor musculature. Correspondingly, epidemiological studies have demonstrated that the incidence of pelvic floor disorders increases with age (45). It was also reported that increased apoptosis of fibroblasts and extracellular matrix remodeling is apparent in older women (46). Additionally, it has been shown that pelvic striated muscle mass decreases gradually as women age (47). These age-related changes also contribute to a higher likelihood of uterine inertia and prolonged labor during vaginal delivery among older mothers, thereby further elevating the risk of POP (48). Collectively, these findings underscore the importance of optimizing the timing of childbirth, suggesting that advanced maternal age should be carefully managed to prevent POP.

In addition, heavy physical labor has been reported as a potential risk factor for pelvic organ prolapse (POP) (44, 49). This may be attributed to the impact of activities such as prolonged heavy lifting on pelvic ligaments and supportive structures (1). The present study broadly categorized participants’ occupations into blue-collar work, white-collar work, and housewives. In our risk factor analysis, blue-collar workers did not demonstrate a significantly higher risk of POP compared with white-collar workers, whereas being a housewife appeared to be a protective factor. A possible explanation is that, although the physical intensity of housework is difficult to quantify, in our region, housewives may benefit from higher household income, better perinatal care, and more adequate postpartum rehabilitation than working women. Nevertheless, these factors were not specifically assessed in the present study and warrant further investigation in future research.

There are several strengths and limitations in this study. One of its major strengths lies in the relatively large sample size, which enhances the statistical power and applicability of the findings within the studied population and our focus on the postpartum women and obstetric features. However, the retrospective design and single-center setting may introduce selection bias and limit the generalizability of the results. Second, this study only included the 6th postpartum week follow-up, the absence of long-term follow-up data restricts our ability to evaluate the persistence or progression of POP over time. Longer and detailed follow-up durations may provide more valuable clinical perspectives. While follow-up within the first postpartum year and from 1 to 5 years can elucidate short-term postpartum pelvic floor recovery and guide rehabilitation methods, follow-up extending to ten or even twenty years into the perimenopausal and postmenopausal periods would help clarify the impact of perinatal factors on later-life POP and contribute to refining perinatal management strategies to improve long-term outcomes. Future multicenter, prospective cohort studies with extended postpartum follow-up and more detailed follow-up assessments are warranted to validate these findings and to better understand the temporal trajectory of pelvic floor recovery and dysfunction.

## Conclusion

A relatively high incidence of POP, primarily stage I, was observed in our study population. Vaginal delivery, multiparity, advanced maternal age, and high pregestational BMI were identified as significant risk factors for POP. Optimizing multiple aspects of preconception preparation and obstetric management, such as careful management of pregnancy at advanced maternal age, weight control while ensuring adequate maternal and fetal nutrition, appropriate intrapartum monitoring, and cautious selection of delivery mode, may help reduce the risk of developing POP.

## Data Availability

The raw data supporting the conclusions of this article will be made available by the authors, without undue reservation.
